# Association of Serum Uric Acid and Coronary Artery Disease in Premenopausal Women

**DOI:** 10.1371/journal.pone.0106130

**Published:** 2014-09-03

**Authors:** Jian-wei Zhang, Ling-jie He, Shu-jun Cao, Qing Yang, Shi-wei Yang, Yu-jie Zhou

**Affiliations:** 1 Department of Cardiology, Beijing Anzhen Hospital, Capital Medical University, Beijing Institute of Heart Lung and Blood Vessel Disease, The Key Laboratory of Remodeling-related Cardiovascular Disease, Ministry of Education, Beijing, China; 2 Department of Emergency, Beijing Friendship Hospital, Capital Medical University, Beijing, China; 3 Department of Cardiology, Beijing Daxing Hospital, Capital Medical University, Beijing, China; Shenzhen Institutes of Advanced Technology, China

## Abstract

**Objective:**

To date, no study in the published literature has investigated the role of various serum uric acid (SUA) concentrations in the development of angiographically-proven coronary artery disease (CAD) in premenopausal women. Therefore, the aim of this study was to investigate the role SUA levels may play in the prevalence, severity, and prognosis of CAD in premenopausal women.

**Methods:**

This cross-sectional retrospective study included 607 premenopausal women who had undergone coronary angiography. The CAD diagnosis was based upon stenosis affecting ≥50% of the luminal diameter. Association of the SUA levels with CAD prevalence, severity, and clinical outcomes were assessed by statistical analysis.

**Results:**

In total, 369 (60.8%) of the patients were diagnosed with CAD. The CAD patients had significantly higher SUA levels than those without CAD (5.3±1.9 vs. 4.8±1.7 mg/dL, *P* = 0.001). The SUA levels were found to be significantly associated with CAD prevalence (*P* = 0.013). Patients with higher levels of SUA also showed increased rates of multivessel disease and composite end-points, such as major adverse cardiac events. Furthermore, multivariate analysis identified abnormally high levels of uric acid (hyperuricemia) as an independent risk factor for CAD (OR 1.51 (1.11–2.53), *P*<0.05).

**Conclusions:**

The SUA levels are significantly associated with the prevalence of CAD. The SUA levels may be a predictor for incidence of major cardiovascular events in premenopausal women.

## Introduction

Menopausal women are at a greater risk of developing coronary artery disease (CAD) than their premenopausal counterparts, a result of the loss of hormone protection that accompanies menopause [1]–[3]. Recently, however, there has been an increase in the number of premenopausal women suffering from angina and myocardial infarction [4]. Due to the scarcity of data available for premenopausal women diagnosed with CAD, little is known of the characteristics, mechanism, prognosis, and risk factors of these patients.

Hyperuricemia, an abnormally high level of uric acid in the blood, was identified as a novel risk factor for the development of CAD [5]. Levels of uric acid, the final product of nucleic acid metabolism, are controlled by a multitude of different factors, including dietary intake, renal excretion, and rate of cell turnover. However, the role of uric acid in CAD remains largely unknown. Recent studies have demonstrated an independent association between serum uric acid (SUA) and CAD [6]–[7]. Moreover, several studies have also demonstrated a strong association of SUA levels with cardiovascular disease (CVD) in women, as compared with their male counterparts [5], [8], [9]. These studies, however, did not evaluate the relationship between levels of SUA and incidence of CAD in premenopausal women. Hence, our study was designed to investigate the role SUA may play in the prevalence, severity, and prognosis of CAD in premenopausal women.

## Methods

### Study design and patient population

A total of 607 premenopausal women, from three major hospitals in Beijing, China (Beijing Anzhen Hospital, Beijing Daxing Hospital, and Beijing Friendship Hospital, Capital Medical University), were included in this study. Women were considered premenopausal if they were between 20 and 50 years of age with a history of regular menstruation of 3–7 days every 22 to 35 days. All of the women had undergone coronary angiography for the first time to address the chest discomfort they were experiencing at the time. Our study obtained the approval from Ethics Committee at the following institutions: Beijing Anzhen Hospital, Beijing Daxing Hospital, and Beijing Friendship Hospital, Capital Medical University. Written informed consent was obtained from each participant or their legal representatives during hospitalization. This study was conducted from January 2007 to May 2012. The data for the study participants were stratified for cross-sectional comparison of patients with and without angiographically-confirmed CAD.

During the study, all of the study’s participants had their blood samples taken on the first day of admission, which was used for SUA analysis by automated biochemical analyzer (Hitachi 747 Biochemical Analyzer; Tokyo, Japan). Outcome/survival data were collected by telephone surveys once a year, for an average of 3.5 years after the initial admission. A total of 13 of the original CAD patients were lost to follow-up due to change in contact information or residence. Based on previously published guidelines, those participants whose SUA level was >6.0 mg/dL were diagnosed with hyperuricemia [4].

### Definition of metabolic syndrome (MS)

The International Diabetes Federation definition of the metabolic syndrome (MS) was used to diagnose participants of the study with this syndrome. Participants had to have a central obesity defined as a waist circumference of ≥80 cm in women based on the Asia-Pacific consensus. In addition, two or more of the following criteria had to be matched: 1) a triglyceride level of ≥1.69 mmol/L (150 mg/dL), 2) an HDL-C level of <1.29 mmol/L (50 mg/dL) in women, 3) systolic/diastolic blood pressure of ≥130/85 mm Hg or previously diagnosed hypertension, and 4) the fasting plasma glucose level of ≥5.6 mmol/L (100 mg/dL) or a previous diagnosis of type-2 diabetes mellitus.

### Coronary angiography

The interventional cardiologist assessment was performed using the standard Judkins technique of angiography. Luminal diameter narrowing of ≥50% in any of the major epicardial coronary arteries was considered sufficient for CAD diagnosis. Participants were assessed during the initial coronary angiography for the presence of multivessel disease, defined as the presence of ≥2 major epicardial coronary arteries or their major branches with stenosis of ≥50%.

### Exclusion criteria

Potential candidates for study enrollment were excluded according to the following parameters: previous history of diuretics usage for antihypertensive medication, cardiomyopathy, valvular heart disease, cardiac surgery, aortic dissection, acute myocardial infarction, or renal insufficiency. In addition, any patients with previously diagnosed CAD were excluded from the study.

### Study end points and definitions in follow-up

Major adverse cardiovascular events (MACE) were cardiac mortality, angina with electrocardiogram changes, acute myocardial infarction, and repeated revascularization.

### Statistical analysis

Categorical data were presented as frequencies (percentages), while continuous data were presented as a mean value ± standard deviation. Differences between the means were compared by unpaired t-test when the variables showed normal distribution or by the Mann-Whitney U test when they did not. For the comparison of categorical variables, either the Chi-square test or the Fisher’s exact test was used. The significant variables in the univariate analysis were introduced in a multivariate logistical regression model to obtain the predictive variables for CAD. The receiver-operating characteristic (ROC) curve analysis was performed to define the sensitivity and specificity of SUA as a discriminator of CAD prevalence. A *P* value of <0.05 (two-sided) was considered significant. Data were analyzed with the SPSS statistical software, version 21.0 (Chicago, Illinois, USA).

## Results

Based on both the inclusion and exclusion criteria, 607 premenopausal women who underwent coronary angiography for the diagnosis or exclusion of CAD were enrolled in the study. Coronary angiographic test results were used to stratify the study participants into a CAD group and a non-CAD group. Baseline characteristics of study subjects are presented in Table 1. The angiographic test diagnosed 369 (60.8%) patients with CAD (≥50% cardiac artery narrowing); the remaining 238 (39.2%) patients composed the non-CAD group.

**Table 1:** Comparison of clinical findings between the non-CAD and CAD groups.

Factors	CAD group(n = 369)	Non-CAD group(n = 238)	t/χ^2^	*P*
Age (years)	42.5±8.6	41.8±9.1	0.957	0.339
WBC counts (10^9^/L)	7.2±2.1	7.0±1.9	1.545	0.123
Serum creatinine (mmol/L)	63.4±14.1	64.2±20.2	−0.574	0.566
hs-CRP (mg/L)	3.48±2.15	3.04±1.92	2.565	0.011
Triglyceride (mg/dL)	161±97	146±106	1.793	0.074
HDL-C (mg/dL)	43±15	46±13	−2.532	0.009
LDL-C (mg/dL)	108.4±46.5	100.6±42.7	2.083	0.038
Total cholesterol (mg/dL)	192.1±62.7	185.9±56.8	1.234	0.218
Uric acid (mg/dL)	5.3±1.9	4.8±1.7	3.297	0.001
Fasting blood sugar (mmol/L)	6.3±3.1	5.8±1.9	2.232	0.014
BMI (kg/m^2^)	24.7±4.2	25.3±4.6	−1.655	0.096
Left ventricular EF %	64.3±8.1	63.2±7.8	1.657	0.098
Hyperuricemia, n (%)	86 (23.3)	38 (16.0)	4.795	0.029
Traditional coronary risk factor, n (%)				
Hypercholesterolemia	128 (34.7)	55 (23.1)	9.211	0.002
Hypertension	130 (35.2)	81 (34.0)	0.091	0.762
Current smoking	25 (6.8)	17 (7.1)	0.031	0.862
Family history	58 (15.7)	30 (12.6)	1.131	0.288
Obesity	75 (20.3)	45 (18.9)	0.183	0.669
Diabetes mellitus	75 (20.3)	45 (18.9)	0.183	0.669
Metabolic syndrome	121 (32.8)	58 (24.4)	4.935	0.026

Data given as mean ± SD or n (%).

CAD, coronary artery disease; BMI, body mass index; EF, ejection fraction; HDL-C, high density lipoprotein cholesterol; hs-CRP, high-sensitivity C-reactive protein; LDL-C, low-density lipoprotein cholesterol; WBC, white blood cell.

The frequencies of hyperuricemia in the CAD and non-CAD groups were 23.3% and 16%, respectively. Both hypercholesterolemia and MS were more prevalent in the CAD group than in the non-CAD group (34.7% vs. 23.1% and 32.8% vs. 24.4%, respectively). Furthermore, the CAD group had a more extensive elevation in high-sensitivity C-reactive protein (hs-CRP), greater levels of low-density lipoprotein-cholesterol (LDL-C) and fasting blood sugar, lower level of HDL-C, and a higher level of SUA (5.3±1.9 vs. 4.8±1.7, *P* = 0.001). The other potential risk factors analyzed (age, triglyceride level, total cholesterol level, body mass index, hypertension, smoking, family history, diabetes mellitus, and obesity) were not associated with CAD in our particular group of coronary angiography newly-diagnosed CAD premenopausal women.

### Clinical value of SUA

**Figure 1:**
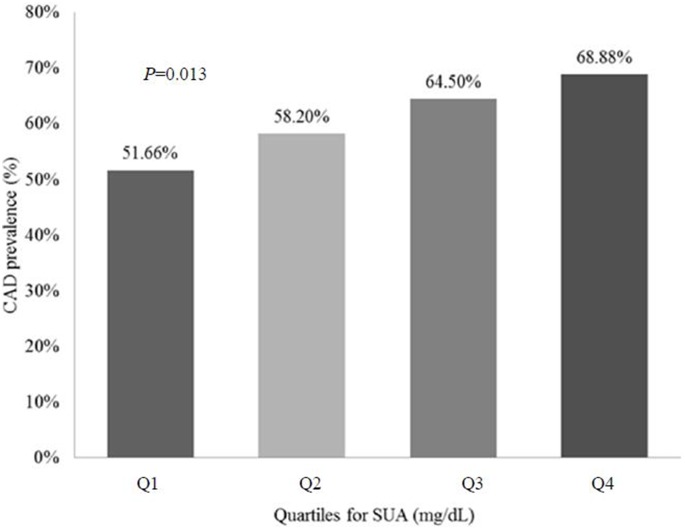
Prevalence of coronary artery disease in premenopausal women as a function of serum uric acid levels.

The SUA quartiles used in this study were as follows: Q1, 1.74–3.58 mg/dL; Q2, 3.59–4.72 mg/dL; Q3, 4.73–5.85 mg/dL; Q4, 5.86–10.52 mg/dL. Based on the univariate analysis, 51.66% of the participants with SUA levels in Q1 had CAD. The patients with levels of SUA in Q2, Q3 and Q4, who also had CAD represented 58.17%, 64.47% and 68.87%, respectively (*P* = 0.04) (Fig. 1 and Table 2). Collectively, these data suggest that increased SUA levels are associated with increasing prevalence of CAD.

**Table 2:** Comparison of quartile levels of serum uric acid between the CAD group and the non-CAD group.

Quartiles	CAD group (n = 369)	Non-CAD group (n = 238)	χ^2^	*P*
Q1: 1.74–3.58 mg/dL	78 (51.7)	73 (48.3)	10.732	0.013
Q2: 3.59–4.72 mg/dL	89 (58.2)	64 (41.8)		
Q3: 4.73–5.85 mg/dL	98 (64.5)	54 (35.5)		
Q4: 5.86–10.52 mg/dL	104 (68.9)	47 (31.1)		

Data given as n (%).

Univariate analysis showed that the risk factors of hs-CRP, LDL-C, fasting blood sugar, hypercholesterolemia, MS, and hyperuricemia played a significant role in CAD (*P*<0.05). In comparison, the traditional coronary risk factors of hypertension, obesity, and diabetes mellitus were not found to be significantly or independently related to CAD.

**Figure 2:**
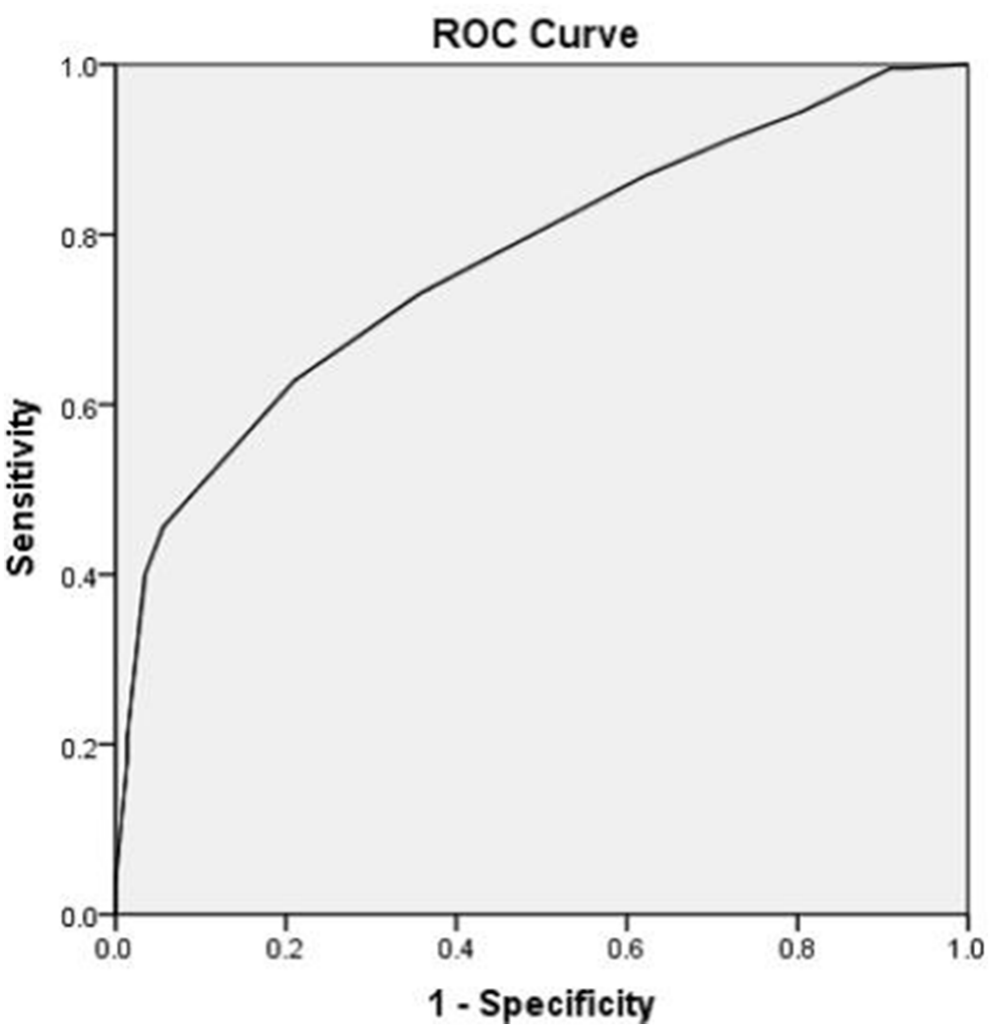
The receiver operating characteristics curve to define the sensitivity and specificity of SUA as a discriminator of CAD prevalence in premenopausal women.

The logistic regression analysis model of CAD risk factors further showed that hs-CRP, LDL-C, hypercholesterolemia, and hyperuricemia were all significant risk factors of CAD (*P*<0.05) (Table 3). The ROC curve analysis showed that the SUA sensitivity as a discriminator of CAD prevalence was 79.0% while the specificity was 62.8%. The area under the curve was 0.770 (95% CI: 0.696, 0.855) (Figure 2).

**Table 3:** Logistic regression analysis model of different CAD risk factors.

Variables	Univariate analysis	Multivariate analysis	
	OR (95% *CI*)	Full model	Final model
		OR (95% *CI*)	OR (95% *CI*)
Age (years)	1.08 (0.85–1.57)	1.10 (0.92–1.71)	
hs-CRP (mg/L)	1.57 (1.13–2.02)*	1.31 (1.03–2.84)*	1.29 (1.01–2.57)*
HDL-C (mg/dL)	0.81 (0.71–0.94)*	0.84 (0.66–1.07)	
LDL-C (mg/dL)	1.66 (1.31–2.21)*	1.49 (1.11–2.35)*	1.21 (1.04–2.15)*
Fasting blood sugar (mmol/L)	1.27 (1.08–2.11)*	1.17 (0.91–2.01)	
Hypercholesterolemia			
Yes	1.77 (1.22–2.56)*	1.64 (1.14–2.32)*	1.34 (1.07–2.12)*
No	1.00	1.00	
Hypertension			
Yes	1.05 (0.75–1.45)	0.89 (0.68–1.28)	
No	1.00	1.00	
Obesity			
Yes	1.09 (0.72–1.65)	1.01 (0.65–1.45)	
No			
Metabolic syndrome			
Yes	1.51 (1.05–2.19)*	1.27 (0.97–1.96)	
No	1.00	1.00	
Hyperuricemia			
Yes	1.60 (1.05–2.44)*	1.44 (1.01–2.12)*	1.51 (1.11–2.53)*
No	1.00	1.00	

Note: CAD, coronary artery disease; CI, confidence interval; HDL-C, high density lipoprotein cholesterol; hs-CRP, high-sensitivity C-reactive protein; LDL-C, low-density lipoprotein cholesterol; OR, odds ratio.

*P<0.05.

The angiographic findings, which indicated involvement of a single vessel, double vessels, triple vessel, main stem, and multivessel, were assessed for the women with CAD according to their SUA quartile (Table 4). In addition, follow-up results regarding cardiac mortality, angina, acute myocardial infarction, repeated revascularization, and composite MACE were assessed for all of the women according to their SUA quartile (Table 4). Analysis of the angiographic findings revealed that events of multivessel disease increased as the concentration of SUA increased (Q1: 34.8%, Q2: 41.9%, Q3: 48.9%, Q4: 56.5%; *P* = 0.022). In contrast, single vessel disease events decreased as the concentration of SUA increased (Q1: 64.1%, Q2: 55.9%, Q3: 46.0%, Q4: 40.0%; *P* = 0.036). Analysis of the follow-up results showed that patients in Q4 had a higher rate of composite MACE compared to all other three quartiles (Q1: 15.6%, Q2: 22.5%, Q3: 23.9%, Q4: 33.7%; *P* = 0.041). All other parameters measured in the follow-up period showed no strong association with CAD across the four quartiles.

**Table 4:** Angiographical findings and follow-up results based on SUA levels in CAD patients.

	Q1 1.74–3.58 mg/dL (n = 92)	Q2 3.59–4.72 mg/dL (n = 93)	Q3 4.73–5.85 mg/dL (n = 92)	Q4 5.86–10.52 mg/dL (n = 92)	χ^2^	*P*
**Angiographical findings of vessel involvement**						
Single vessel	59 (64.1)	52 (55.9)	46 (50.0)	40 (43.5)	8.560	0.036
Double vessel	16 (17.4)	20 (21.5)	22 (23.9)	25 (27.2)	2.688	0.442
Triple vessel	14 (15.2)	16 (17.2)	18 (19.6)	21 (22.8)	1.946	0.584
Main stem	3 (3.3)	5 (5.4)	6 (6.5)	6 (6.5)	1.273	0.736
Multivessel	32 (34.8)	39 (41.9)	45 (48.9)	52 (56.5)	9.676	0.022
**Follow-up results**						
Cardiac mortality	1 (1.1)	2 (2.3)	2 (2.3)	3 (3.4)	1.025	0.791
Angina	8 (8.9)	10 (11.2)	12 (13.6)	16 (18.0)	3.522	0.309
AMI	2 (2.2)	3 (3.4)	3 (3.4)	4 (4.5)	0.691	0.871
Repeated revascularization	5 (5.6)	7 (7.9)	8 (9.1)	9 (10.1)	1.320	0.710
Composite MACE	14 (15.6)	20 (22.5)	21 (23.9)	30 (33.7)	8.052	0.041

Data given as n (%). AMI, acute myocardial infarction; MACE, major adverse cardiac event.

## Discussion

The principal finding of our study was the demonstration that SUA levels are markedly related to the prevalence of CAD in premenopausal women. Our results strongly suggest that hyperuricemia is an independent risk factor for CAD in this specific target group. The current literature does not provide a consensus on the SUA role in the CAD development. However, a study published in 2013 suggested that SUA was not predictive of either CVD or CVD death and that SUA did not increase patient’s risk in conjunction with the traditional CVD risk factors [10]. However, multiple studies have shown that SUA levels are associated with risk of CAD and adverse cardiac events, especially in women. Calvo and colleagues reported a study of postmenopausal Caucasian and Filipino women, in which they identified SUA as a marker of CAD progression [8]. Similarly, Lin and colleagues showed that in a 3.1 year median follow-up elevated SUA could predict the rate of both cardiac and all-cause mortality among patients with angiographically-proven CAD [11]. Ndrepepa and colleagues showed that in subjects with Type 2 diabetes mellitus and confirmed CAD, elevated SUA could predict mortality independently of known traditional cardiovascular risk factors [7].

A meta-analysis of the hyperuricemia and CAD showed strong association between hyperuricemia and an increased risk of CAD prevalence, independently of traditional CHD risk factors [5]. Further analysis of the subgroups did not identify a significant association between hyperuricemia and CHD prevalence/mortality in men, but did show an increased risk for CHD mortality in women [5]. Another study showed that increased SUA levels were predictive of an increased risk of both cardiac and all-cause mortality, independent of traditional cardiovascular risk factors and that these associations followed a ‘J-shaped’ pattern [6]. Finally, this study also showed that the strongest association between SUA and mortality occurred in women and patients without arterial hypertension [6]. The well-recognized difference in cardiovascular risk profile of women, compared to men, may partly explain the stronger association between hyperuricemia and cardiovascular events in women.

Compared to their male counterparts, premenopausal women have lower SUA levels [12], [13] and less cardiovascular risk factors. Hence, it is not clear why, in premenopausal patients with CAD, estrogen is not able to exert its cardiovascular protective function. One explanation suggests that the excessive inflammation, estrogen receptor deficit, and gene variation may be potential factors affecting the estrogen protective function [14], [15]. Moreover, components associated with MS may modulate the platelet numbers, receptor-activated platelet secretory functions, and cellular activation of the vascular wall contributing to atherogenesis [16] while anti-atherogenic and anti-inflammatory properties of HDL may be diminished under certain inflammatory conditions [17]–[22]. The data from our current study showed a strong association between high level of SUA and development of multivessel disease and composite MACE. Findings from a previous study showed that the premenopausal CAD patients, more frequently presented with single vessel narrowing than their postmenopausal CAD counterparts (43.2% vs. 26.9%), had more severe lesions (≥90%) in the left main coronary artery (2.9% vs. 1.1%) and proximal left anterior descending artery (28.2% vs. 16.6%) [4]. These patients also had more triple vessel involvement (33.8% vs. 26.9%) [4]. Our study confirmed that in premenopausal women the rate of a single vessel attained was 53.4%. However, following the increase in SUA levels the rate of multivessel diseases was elevated. Moreover, ROC curve analysis indicated that SUA may be developed as a clinical discriminator of CAD prevalence for use in screening premenopausal women.

For many years, SUA has been used in clinical practice as a marker of several metabolic disturbances. It has been generally agreed on that MS confers increased risk for CAD and poor long-term prognosis [23]–[24]. Due to its association with CAD, SUA could be used as a marker for a) cardiovascular risk factor burden, b) endothelial dysfunction, c) oxidative stress, d) diabetes, and e) hypertension [25], [26], all of which are important predictors of CAD. In addition to hyperuricemia, our study results have shown that premenopausal women with CAD had higher incidents of hypercholesterolemia, MS, and increase in levels of hs-CRP, LDL-C and fasting blood sugar. Furthermore, levels of HDL-C were lower in these patients compared to non-CAD patients, potentially increasing their risk of MACE in during follow-up evaluations.

Our results further strengthen the notion that with high levels of SUA, premenopausal women have a greater chance of developing a composite MACE. However, currently available models used for the study of pathophysiological mechanisms of uric acid do not adequately explain the relationship between hyperuricemia and CAD. Soluble uric acid has been shown to act as a pro-oxidant, as well as facilitator of free radical production [27]. Uric acid can crystallize resulting in the formation of monosodium urate crystals (MSU), which precipitate in various tissues, triggering a local inflammatory response. The mechanistic actions involving MSU have been shown to be intimately involved in the pathology of CAD [27]. Moreover, SUA can stimulate oxidative stress, induce endothelial dysfunction, inflammation, and vasoconstriction [28]. These mechanisms may potentially contribute to the development of CAD observed in premenopausal patients with hyperuricemia.

SUA is associated with other risk factors. Therefore, decreasing SUA levels by administrating statin [29] in combination with other measures may reduce the risk of CAD. Further research is necessary to better understand the biological role of uric acid in CAD, potentially providing new therapeutic targets for the prevention and treatment of CAD in premenopausal women.

### Study limitations

There are several limitations to this study. First, this was not a randomized trial, but a retrospective evaluation. Second, we had a relatively few premenopausal women who underwent coronary angiography. Third, estrogen levels were not measured in the study. Hence, the results of the present study should be interpreted with caution.

### Conclusions

The prevalence of CAD in premenopausal women is significantly related to SUA levels. Hyperuricemia was identified as an independent risk factor for CAD after adjusting for potential confounding variables. SUA levels, measured at the time of positive angiographic CAD diagnosis, may represent a useful clinical predictor of incidence of major cardiovascular events in premenopausal women over a 3.5-year follow-up period.
